# *TAZing down metabolic mayhem*: siRNAs against liver inflammation and fibrosis in humanized mice

**DOI:** 10.1016/j.omtm.2023.101182

**Published:** 2024-01-01

**Authors:** Julian Weihs, Milad Rezvani

**Affiliations:** 1 Charité – Charité University Medicine Berlin, Corporate Member of Freie Universität Berlin and Humboldt Universität zu Berlin, Department of Pediatric Gastroenterology, Nephrology and Metabolic Medicine, Berlin, Germany; 2 Berlin Institute of Health (Center for Regenerative Therapies, Clinician-Scientist Program), Berlin, Germany; 3 Division of Gastroenterology, Hepatology and Nutrition, Cincinnati Children’s Hospital Medical Center, Cincinnati, OH 45229, USA

Metabolic dysfunction-associated steatotic liver disease (MASLD)—generally the consequence of Western lifestyle and genetic predispositions—is the most common cause of liver fibrosis. The spectrum of disease progression ranges from steatosis to mixed inflammatory cell infiltration (steatohepatitis) and, eventually, cirrhosis. This process also rewires the transcriptional network within hepatocytes—the main parenchymal cell type. Thereby, usually silent developmental pathways reemerge in hepatocytes, including the YAP-TAZ (Hippo) pathway. Importantly, TAZ induces Indian Hedgehog pathway, which causes pro-fibrotic signaling. To break this cycle, Wang et al.^1^ join the current “gold rush” for an effective therapy against MASLD—the most common liver disease worldwide.^1^ To overcome limitations related to inter-species differences, their team elegantly demonstrates anti-fibro-inflammatory effects by silencing *TAZ* in steatotic human hepatocytes in highly chimeric mice using small interfering RNA (siRNA) technology.

Mice harboring human hepatocytes carry great potential for advancing gene therapies, as demonstrated for *ex vivo* therapies of classic monogenetic liver diseases.^2^ “Humanized mice,” rooted in immune-deficient strains with mouse hepatocyte injuries, allow human hepatocytes to engraft, repopulate, and substitute mouse hepatocytes. The menu of available mice is long and involves genetic liver injuries as the PiZ allele of SERPINA1 gene (NSG-PiZ mouse), Urokinase-type plasminogen activator (uPA-SCID mouse), and Fumaryl acetoacetate hydrolase knockout (FAH mouse), among others. With the increasing availability of these mice, Wang et al. procured from a commercial vendor a variation of the uPA-SCID mouse model, the cDNA-uPA/SCID mouse with a reportedly more long-lasting humanization capacity.^3^ Proceeding with mice carrying >90% human hepatocytes, their team fed them a high-fat, choline-deficient L-amino acid-defined chow, a diet used for conventional modeling of MASLD. This strategy is reminiscent of a recent study where humanized mice were fed a high-fat diet.^4^ Yet, these two approaches highlight how current humanized MASLD models lack the complexity of a real-world, human Western Diet.

Although MASLD follows a multicellular pathophysiology, hepatocytes have gained increasing attention, with dysregulated lipid metabolism and lipotoxicity affecting primarily hepatocytes. Another group recently harnessed NSG-PiZ humanized mice with a genetic obesity-driven mouse model of MASLD and silenced diacylglycerol O-acyltransferase 2, a key enzyme in hepatocyte triglyceride synthesis.^5^ Overall, this illustrates how in humanized MASLD models, different therapeutic strategies can be identified—ranging from early steatosis modifiers to global transcriptional hepatocyte regulators. Wang et al.’s innovative approach showcases a compelling reduction in fibro-inflammatory infiltration at the intersection of tissue damage and cellular reprogramming. Their utilization of siRNAs conjugated with N-acetyl-galactosamine holds promise, considering recent FDA approvals for hepatocyte-targeted siRNAs. However, the inherent limitations of humanized models persist, e.g., the absence of a syngeneic human immune system. Yet, such extensively humanized models would demand sophisticated infrastructures. Lastly, addressing gender-specific aspects and utilizing diverse human hepatocyte donors would augment the breadth and applicability of these models, considering this study’s focus on a single donor with a PNPLA3 risk variant and male recipient mice.
